# Refinement of a chronic cranial window implant in the rat for longitudinal *in vivo* two–photon fluorescence microscopy of neurovascular function

**DOI:** 10.1038/s41598-019-41966-9

**Published:** 2019-04-02

**Authors:** Margaret M. Koletar, Adrienne Dorr, Mary E. Brown, JoAnne McLaurin, Bojana Stefanovic

**Affiliations:** 1 Department of Medical Biophysics, University of Toronto, 610 University Avenue, Toronto, Ontario M5G 2M9 Canada; 2 Sunnybrook Research Institute, 2075 Bayview Avenue, Toronto, Ontario M4N 3M5 Canada; 3 Department of Laboratory Medicine and Pathobiology, University of Toronto, 1 King’s College Circle, Toronto, Ontario M5S 1A1 Canada

## Abstract

Longitudinal studies using two–photon fluorescence microscopy (TPFM) are critical for facilitating cellular scale imaging of brain morphology and function. Studies have been conducted in the mouse due to their relatively higher transparency and long term patency of a chronic cranial window. Increasing availability of transgenic rat models, and the range of established behavioural paradigms, necessitates development of a chronic preparation for the rat. However, surgical craniotomies in the rat present challenges due to craniotomy closure by wound healing and diminished image quality due to inflammation, restricting most rat TPFM experiments to acute preparations. Long-term patency is enabled by employing sterile surgical technique, minimization of trauma with precise tissue handling during surgery, judicious selection of the size and placement of the craniotomy, diligent monitoring of animal physiology and support throughout the surgery, and modification of the home cage for long-term preservation of cranial implants. Immunohistochemical analysis employing the glial fibrillary acidic protein (GFAP) and ionized calcium-binding adaptor molecule-1 (Iba-1) showed activation and recruitment of astrocytes and microglia/macrophages directly inferior to the cranial window at one week after surgery, with more diffuse response in deeper cortical layers at two weeks, and amelioration around four weeks post craniotomy. TPFM was conducted up to 14 weeks post craniotomy, reaching cortical depths of 400 µm to 600 µm at most time-points. The rate of signal decay with increasing depth and maximum cortical depth attained had greater variation between individual rats at a single time-point than within a rat across time.

## Introduction

Longitudinal *in vivo* brain studies have become widely recognized as critical for progress in neuroscience. The ability to scrutinize induction, progression, and resolution of a (patho)physiological process within a single animal can greatly increase sensitivity, allow for reduction of the number of experimental animals used, and is of particular interest in awake imaging experiments where effort is expended upfront into animal acclimatization and training. Longitudinal two–photon fluorescence microscopy (TPFM) in particular, has provided unprecedented cellular scale data on brain morphology and function. Most longitudinal TPFM experiments to date have been performed in mice. The reduced skull density and translucent meningeal tissues in combination with less opaque cortical tissue allow a thinned skull preparation, providing adequate light penetration while minimizing trauma to underlying parenchyma^1–3^. Modification to the thinned skull with the addition of a glass coverslip stabilizing and preserving the thinned region enhances longitudinal integrity of the window^2,4^. Craniotomies installed with a glass coverslip on the skull surface^5–9^, or thinning of the skull around the craniotomy such that the glass coverslip rests on the dura^10^ have been described in detail. Chronic implants in mice have also incorporated specialized chambers for super-perfusion^11^, silicon injection ports^12^, and removable window techniques for subsequent tissue transplants^13^.

There is, however, growing interest in rat models, due to recent development of transgenic rat lines as well as the wide range of behavioural and, in particular, cognitive testing batteries that have been well-characterized in the rat. A chronic craniotomy in the rat has been challenging and rarely successful in meeting the TPFM imaging requirements^3^. Inherent challenges with the surgical procedure result in damage from bleeding^14,15^, exposure of the pial surface to the environment^16^, inflammation^6,7,17,18^, and distortion of parenchymal and vascular architecture by edema. The rat models are more difficult than the mice due to mechanical damage to underlying tissue while drilling through a thicker skull, and the necessity for removing meningeal layers. These induce prolonged inflammation and acceleration of wound healing mechanisms. Subsequent opacity and closure of the window by infiltrating fibrotic tissue diminishes light penetration. These challenges have resulted in TPFM investigations in the rat being largely restricted to acute preparations.

Notwithstanding, concerted efforts to allow chronic TPFM in the rat are on the rise. Scott *et al*.^19^, described successful implantation of a chronic window in a rat facilitating TPFM imaging for weeks after surgery. They conducted imaging in behaviourally acclimatized awake rats following dural removal and implantation of a specialized head mount to allow reproducible alignment of the cranial window under the microscope’s objective. TPFM in these awake restrained rats, transfected with a fluorescent Calcium indicator, allowed imaging at 150–300 µm below cortical surface up to 4 weeks after surgery, with some craniotomies remaining patent beyond the experimental time course. A more recent report by Heo *et al*.^20^, demonstrated a chronic implant in the rat and mouse, implementing a cranial window with a silicone (polydimethylsiloxane) covering instead of a glass coverslip. The purpose was to accommodate either electrophysiological recordings or micro-injections through a glass pipette, for image acquisition with either optical recording of intrinsic signal (rat), or TPFM (mouse). In the rat, cranial windows were patent up to at least 10 weeks after surgery, while mice remained patent for months.

In this study, we endeavoured to provide a more generalized description of techniques for chronic TPFM in the rat, building upon prior work in the mouse^3,5,7,9–13,21^. The key determinants of long-term transparency for successful TPFM include minimization of damage during removal of the skull bone, precise excision of the meningeal layers, optimization of craniotomy size and placement of the glass coverslip, close physiological monitoring of the animal during surgery, and modification of the home cage for long-term preservation of the implant. Success of this technique is demonstrated by long-term post-surgical recovery and deep *in vivo* TPFM, with progressive attenuation of neuro-inflammation in the region of the craniotomy. TPFM was performed biweekly at 400–600 µm below the cortical surface over a 14 week time course.

## Methods

### Animals

The study was conducted using male Sprague Dawley rats acquired from Charles River Canada. The optimal size of the rat was identified based on ease of handling and window transparency, as 250 to 300 grams or 8 to 10 week old. At this age, a larger craniotomy could be accommodated, while still occurring prior to full maturity of the skull bone. This size was also more convenient for daily handling for behavioural training, and allowed ready healing of the implant onto the calvaria. Animals were housed on a ventilated rack under a 12 hour light:dark cycle with food and water ad libitum. Five rats were used for development of the technique, and refinement of the sterile surgery protocol (rat CC1-CC5). Seven rats received chronic craniotomy implants for establishing longitudinal TPFM (rat CC6-CC12). TPFM was conducted at 2, 4, 6, 8, 10, 12 and/or 14 weeks after surgery. Four of the 7 rats were imaged at a subset of time-points, so as to minimize stress from frequent anesthetic induction. Two of the 7 rats underwent imaging at all seven time-points between 2 to 14 weeks. One rat (CC8) succumbed during the first TPFM session at 2 weeks. On the day of imaging, two stacks of images parallel to the cortical surface were acquired, each in a separate location within the cranial window. The imaging location within the cranial window was varied on subsequent imaging days to reduce potential tissue damage by repeated laser exposure. Three additional rats (rat CC13–15) received chronic craniotomy surgery for immunohistochemical analysis at 1, 2 or 4 weeks after surgery. Supplementary Table S1 summarizes the number of rats utilized for development of the surgical technique. All experimental procedures in this study were approved by, and performed in accordance with the regulations established by the Animal Care Committee of the Sunnybrook Research Institute, which adheres to the Policies and Guidelines of the Canadian Council on Animal Care (CCAC) and meets all the requirements of the Provincial statute of Ontario, Animals for Research Act as well as those of the Federal Health of Animals Act, and the International Council for Laboratory Animal Science (ICLAS).

### Surgery

**Figure 1:**
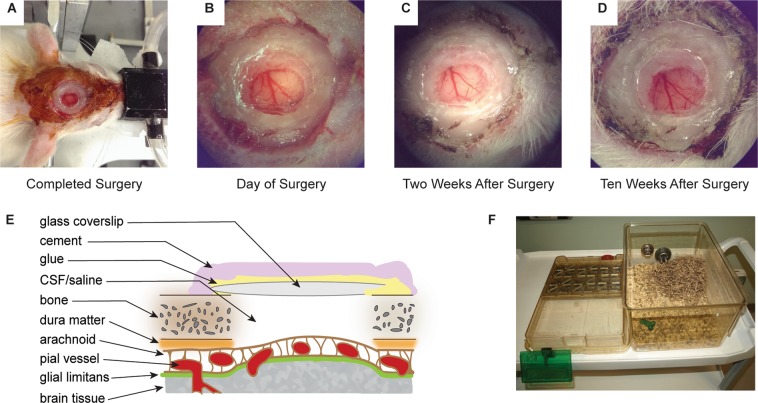
Surgical implant procedure of a chronic craniotomy in a rat. (**A**) Completed surgery in a rat just prior to recovery from anesthesia; (**B**) Close-up of the completed craniotomy implant on the day of surgery; (**C**) Two weeks after surgery; and (**D**) Ten weeks after surgery. (**E**) Cross-sectional rendering of the craniotomy and tissue layers affected in the surgical procedure. The technique used for placement of the glass coverslip (skull surface, thinned skull, or adjacent to the pial surface) will modify application of cementing compound, the degree of distortion of pial surface, and the extent of inflammation below the coverslip. Coverslip placement on skull surface is indicated (not to scale). (**F**) Example of a modified traditional rat cage showing the elimination of overhead obstructions that could damage the implant (e.g. wire food rack, PVC tube, etc.), and with additional enrichment (shredded paper bedding, chew toy, etc.). Photographs (**B–D**) were captured through the surgical stereoscope eyepiece at 6x magnification.

Implanting a chronic cranial window requires familiarization with sterile surgical techniques and proficiency in basic surgical skills. Gentle handling with precise manipulations of tissues minimizes trauma and facilitates establishment of the long-term cranial window. Supplies and agents employed are summarized in Supplementary Table S2. A presentation of a successful cranial window is illustrated in Fig. 1A–D. Knowledge of tissue anatomy and effects from surgical manipulation is essential preparation. Figure 1E represents the fundamental components described, for reference. Actual results for each cranial implant will depend on the technique used for placement of the coverslip, and other modifications necessary for the experimental paradigm.

The rat was anesthetized with 5% inhalant isoflurane in a rodent induction chamber, weighed, transferred to the stereotax (SR-50, Narishige International USA), and secured with the ear bars and incisor bar set for flat skull (3–4 mm). Standard animal surgical room configurations provide Isoflurane with 100% oxygen as the carrier gas. For this study, 2% isoflurane in medical air was delivered via a nose cone and supplemented with oxygen to achieve a total concentration of 30–40% oxygen. Isoflurane acts as a respiratory depressant, decreasing respiratory tidal-volume in a dose dependent manner, leading to hypoxia/hypercapnia, vascular acidosis, vasodilation, and increased cerebral blood flow that may increase intracranial pressure^22^. Providing an increase in inspired oxygen concentration counteracts the anesthetic effects by decreasing end-tidal hypercapnia and cerebral blood flow through vasoconstriction^23^. However, inspiring pure oxygen may induce hyperoxia in free breathing animals. This leads to decreased blood perfusion due to excessive vasoconstriction, resulting in poor tissue oxygenation and hyperoxia/hypocapnia^24^. Limited oxygen enrichment ameliorates negative surgical effects such as bleeding and tissue swelling. Alternatives in anesthesia, such as Propofol, or balanced anesthetic:anxiolytics such as Ketamine:Xylazine, may provide further refinements to minimize bleeding and/or inflammation at the cost of increasing the complexity of anesthesia level titration^25–29^. Monitoring and adjustment of physiological parameters throughout the surgical protocol is invaluable to help ensure the well-being of the animal and increase success rates. A pulse oximeter (MouseOx Plus, Starr Life Sciences Corp, USA) was employed to monitor heart rate, respiration rate, and arterial oxygen saturation, via a hind paw probe. An electric heating pad with a thermorectal feedback probe (Temperature Controller TC-1000, CWE Inc, USA) was employed to maintain the core body temperature at 37 °C. After application of an eye lubricant to prevent corneal desiccation, each rat received hydrating fluids (Lactated Ringer’s Solution, 3 mL, subcutaneously), antibiotic (enrofloxacin, 5 mg/kg, subcutaneously), anti-inflammatory (dexamethasone, 0.1 mg/kg, intraperitoneally) and analgesic (buprenorphine, 0.05 mg/kg, subcutaneously).

The dorsal surface of the head was shaved from the neck up to between the eyes, being careful not to damage the eye lashes or whiskers. The skin was then cleaned with a chlorhexidine soap, isopropyl alcohol swab, and then Betadine Solution (10% iodine) painted over the surface of the skin up to the edge of the fur. A local anesthetic (Marcaine, 7 mg/kg, subcutaneously) was infused around the incision site.

Sterile surgical technique was strictly followed to reduce tissue stress exacerbated by exposure to potential infection and subsequent inflammation. Surgical preparation thus included sterile drapes, instruments and surgical supplies arranged on the table, and cap, mask, sterile gown and gloves. A mid-line incision was made from the base of the skull to the frontal bone between the eyes, approximately 3 cm in length. After retracting the skin (Colibri retractor, Fine Science Tools, Canada) to expose the periosteum, the skull was gently cleaned by blunt dissection followed with a sterile cotton tipped swab. We found that using a scalpel blade to scrape the bone created micro-abrasions and chronic bleeding into or around the craniotomy. Cold saline applied on the skull and tissue induced vasoconstriction and reduced bleeding, in addition to cleaning the skull. Alternatively, cold artificial cerebrospinal fluid (aCSF) may be used in place of saline. While being vigilant not to disturb the eye, the Temporalis muscle was gently separated from the bone along the Temporal ridge so as to prevent interference by the tissue while drilling. Flat skull was achieved adjusting height of bregma with lambda, and two lateral points ±2 mm from sagittal, setting the incisor bar between 3 to 4 mm for each rat. The skull surface was marked using a sterile surgical marker (or the bone scored with a fine tipped #5 Dumont forcep) outlining a 6 mm diameter craniotomy over the somatosensory cortex (AP +2 to −4 mm; ML ±0.5 to ±6 mm). We used a surgical stereoscope^14^ and a hand held high-speed micromotor drill with a round engravers drill bit for manual control of the size and shape of the craniotomy. Beginning with a 1.0 mm drill bit, a circular cut was made within the marked borders of the craniotomy. The drilling was frequently paused for cooling of the skull bone with cold saline, so as to reduce heat damage, edema, and control bleeding around or underneath the skull. The saline rinses were followed by drying with sterile gauze or Kim Wipes. Nearing the inner periosteal and dural layers, the drill bit was changed to a 0.45 mm for fine control. To avoid damage, drilling should not extend beyond the lower margin of the bone (i.e. deep periosteum). A smooth vertical margin of the bone was created around the craniotomy until the center skull piece was released from the edges. Extra vigilance ensured the drill bit never contacted the dura or pial surface. At this point, room temperature saline was used to flush the cranial region.

The skull piece was carefully separated from the skull, using a #5 Dumont forcep. Slow retraction of the skull piece at one edge encouraged separation from the underlying dura. Forceful pulling on the dura tears the arachnoid trabeculae attached to fragile pial vessels and pia matter. Tearing of this tissue releases pro-inflammatory signals, potentiating glial activation in the parenchyma^30,31^. Subdural bleeds lead to clotting and adhesion of the dura to the pial surface. As the skull piece was being lifted upward, another forcep was gently scraped along the underside of the skull piece, with frequent saline flushes to encourage release from the dura. Hemostasis was continuously maintained and the craniotomy was kept moistened throughout the procedure.

Prior to removing the dura, all tissue and bone fragments protruding into the cranial window (including bone dust, debris, periosteal layers) were removed, and craniotomy rinsed clean. Remaining tissue or debris within the window provides a substrate for infiltrating fibroblasts. A 25 gauge needle was bent 45 degrees at the beveled tip using a mosquito haemostat. The dura was visualized using the 25x magnification objective on the surgical stereoscope. The dura was slowly lifted with fine forceps in an area devoid of pial vessels. Gentle retraction was held until the dura separated from the pial surface. The 25 gauge needle was then used to pierce the dura and create a hole, while avoiding any contact with the cortical surface. Precise cutting of the dura adjacent to the bone ensures atraumatic removal of the tissue. Switching to micro-spring scissors, the dura was again retracted and cut close against the edge of the skull bone around the circumference of the window. Excising the dura close to the bone minimizes tissue protruding into the craniotomy. Effort must be made to avoid tearing the dura or contacting the cortical surface with surgical instruments, which potentiates the inflammatory cascade. Diligent hemostasis continued through the procedure.

The craniotomy was closed as soon as bleeding and debris were cleared. A few drops of sterile saline were placed in the craniotomy avoiding excessive spillage over the skull surface. The 8 mm round glass coverslip (World Precision Instruments, USA) was held at a 45 degree angle on one edge of the skull opening and then slowly lowered, eliminating air bubbles. If a bleed occurred, the coverslip was removed and hemostasis reinstated. A 1 mL syringe with a 25 gauge needle was filled with cyanoacrylate glue for accurate delivery around the edge of the coverslip, sufficient to provide contact between the skull and coverslip but prevent wicking underneath the coverslip as it dried. Gentle pressure was applied to hold the coverslip against the skull. The glue was allowed to cure for 10 to 15 minutes before continuing.

Dental cement was used to create a well around the cranial window to accommodate the water immersion objective lens for TPFM. In a sterile glass petri dish, the cement compound was mixed as per manufacturer’s instructions (Ortho-Jet, Lang Dental Manufacturing Co., USA). An alternative that is particularly helpful in sophisticated cranial implants is the light curable dental bonding agent. The light curing cements can eliminate the need for the initial glue stabilizing the coverslip, and provide immediate bond strength onto the skull (RelyX Ultimate #56891, Elipar DeepCure-S #76076, 3 M Dental, Canada). The well was placed around the periphery of the coverslip so as to cover the cyanoacrylate glue and build a wall 1–2 mm high. It was useful to shape the well around the outer circumference of the coverlip, providing space for the objective lens to move within the window. Our preference was not to cover the entire dorsal skull surface with dental cement, as some protocols suggest. This requires a greater amount of skin to be removed and may produce greater tissue irritation from the chronic implant. The cement was allowed 15 minutes to solidify before suturing the skin.

Closure of the surgical site was followed by removal of the tissue retractor, hydrating and cleaning the surrounding tissue with sterile saline, and inspecting the wound to eliminate glue, bone debris, and clotted blood. The skin was retracted around the implant ensuring contact with underlying skull surface. Excess skin was minimally trimmed around the implant to achieve a snug apposition against the side of the cement well and seal the implant. All bleeding from skin or tissue was controlled with mosquito haemostat clamps. The skin was sutured closed using 4–0 absorbable suture (Polysorb P-14, Covidien-Medtronic, USA) while being cautious to maintain normal tension around the eyes. Cyanoacrylate glue or surgical skin glue (Vetbond, 3M Animal Care Products, USA) was applied sparingly between the skin and cement well to anchor the skin onto the skull and prevent growth over the craniotomy (Fig. 1A–D). Antibiotic ointment was applied over the entire shaved area of skin, additional hydrating fluids were administered (Lactated Ringer’s Solution, 3 mL, subcutaneously), and isoflurane was discontinued. The rat was then transferred from the stereotaxic apparatus to a warmed recovery cage until fully mobile, walking, grooming, eating and drinking by its own volition. Post-surgical analgesics (carprofen, 5 mg/kg, subcutaneously) and antibiotics (enrofloxacin, 5 mg/kg, subcutaneously) were administered twice daily for three days as per institutional Standard Operating Procedures.

### Modification to Home Cage

To protect the implant, all overhead obstructions were eliminated from the cage, including wire tops that dispense food or hold water bottles, PVC tubes or huts that provide shelter, etc. Increasing the overhead clearance provided a larger volume of living space for the rat that is also beneficial for the animal’s well-being. We utilized chew toys, occasional treats buried for scavenging, nesting material, shredded paper or soft paper products that did not interfere with the implant (Fig. 1F).

As co-housing rats provides beneficial enrichment, we made every effort to maintain two rats per cage after surgery. Individuals that were singly housed for more than 6 weeks after surgery became difficult to handle. Co-housing required both rats to undergo chronic craniotomy or sham surgery on the same day, and to be recovered together. None of the cranial implants were damaged or compromised from grooming, rough play, or other interactions.

### Immunohistochemistry

An additional three rats received chronic cranial windows and were allowed to recover for 1, 2 or 4 weeks to assess the astrogliosis and microglia/macrophage recruitment in the cranial window region pathologically. The rats were anesthetized with isoflurane, and then transcardially perfused with cold heparinized phosphate buffered saline followed by cold 4% paraformaldehyde (PF101FD, Neuro Technolgies Inc, USA). The caudal aorta along the dorsal thoracic wall was clamped with a haemostat to reduce the volume of perfusate required. Whole brain explants were cryoprotected in 30% sucrose/phosphate buffered saline solution and sectioned on a microtome (Microm HM400, MICROM International, GmbH), producing 40 µm coronal slices across the craniotomy.

Free-floating tissue sections were blocked in goat serum (6210-072, Gibco Thermo Fischer Scientific, USA), incubated overnight in anti-GFAP rabbit (Z0334, Dako, USA) for astrogliosis, or anti-Iba-1 rabbit (019-19741, Wako, USA) for microglia/macrophage recruitment, followed by secondary Alexa Fluor 488 anti-rabbit goat IgG (A11008, Life Technologies, USA) antibodies. Images were acquired using a Zeiss Observer.Z1 (Carl Zeiss Canada Ltd) fluorescent microscope, with ApoTome.2 and Axiocam HR R3 camera, and ZEN 2.5 Pro imaging software.

### Two-photon fluorescence microscopy

Imaging was conducted at two week intervals beginning 2 weeks after surgical implant of the chronic craniotomy, and up to 14 weeks. Each animal was not imaged at every time-point to reduce stress in the individual animal. Prior to each imaging session, the animal was induced with 5% isoflurane in an induction chamber, then transferred to the stereotax (SR-50, Narshige International, USA). For primary assessment of surgical success in this study, we used isoflurane maintained at 1.5 to 2.5% via nose cone. Alternative anesthetics described above are important considerations for acquiring relevant data unique to each experimental design^25–29^. Hydrating fluids (Lactated Ringer’s Solution, 3 mL, subcutaneously) and eye lubricant were administered. Body temperature was maintained at 37 °C by a thermorectal feedback heat pad and temperature controller (TC-1000, CWE Inc, USA). Physiological parameters were monitored continuously by pulse oximetry (MouseOx Plus, Starr Life Sciences Corp, USA). The nonsteriodal anti-inflammatory drug (NSAID) carprofen was administered (5 mg/kg, subcutaneously) as an analgesic and anti-inflammatory to alleviate minor discomfort after TPFM imaging sessions. Under a surgical stereoscope, the cranial window was examined for glass coverslip integrity, purulent infection, cortical edema or deformation, translucent or opaque fibrotic tissue growth (i.e. wound healing), and general appearance of the pial vessels. Fur growth around the implant was trimmed as necessary. The glass coverslip was gently cleaned with an isopropyl alcohol swab to remove debris.

Texas Red fluorescent dextran (Invitrogen, 70 kDa; 12.5 mg/mL in sterile PBS, 20 mg/kg) was administered through a 24 gauge intravenous catheter placed in the lateral tail vein to facilitate imaging of the brain microvasculature. With all longitudinal procedures, sterile technique for placement of the intravenous catheter was strictly followed to minimize trauma to the tail. The rat was then transferred to the microscope stage, positioned under the objective lens, and tilt, rotation, and incisor bar adjusted to ensure pial surface was parallel to the plane of the objective. The dental cement well was filled with clean deionized distilled water. Two locations, within the cranial window on the pial surface, were selected at each time-point, where arteriolar and venular branches penetrated the cortex. Imaging was performed using an FV1000MPE multiphoton laser scanning microscope (Olympus Canada Inc). A Mai Tai Ti:Sapphire tunable laser (690–1040 nm; Newport Corp, USA) was tuned to 900 nm to excite the fluorescent dextran. All imaging employed a 25x, 1.05 NA, 2 mm working distance objective lens (Olympus Canada Inc). An external photomultiplier tube (Hamamatsu Inc, USA) collected red fluorescent emissions (570–620 nm band pass emission filter). A stack of slices parallel to the cortical surface were captured using 0.8 µs/pixel dwell time, 512 × 512 µm field-of-view, every 1.5 µm step in the axial direction (FluoroView FV10-ASW, Olympus Canada Inc). Laser power was corrected for depth to achieve adequate signal-to-noise ratio (SNR) at deeper cortical layers, up to a maximum of 50 mW to minimize tissue damage^32^. Images were thus collected as deep as 700 µm below the cortical surface on some occasions. However, depth was frequently limited by the physical obstruction of the objective lens contacting the glass coverslip or cement well.

### Data analysis

Image data were imported into Imaris software (Imaris x64 9.2.1, Bitplane Inc, USA) for visualization. Maximum intensity projection (MIP) along the axial direction were computed. Slices parallel to the cortical surfaces at the pial surface, 200 µm, 400 µm, and 600 µm depths underwent vessel-wise analysis. Signal intensity (arbitrary units, Gray Scale, ImageJ 1.51n, USA) was measured along the line perpendicular to the long axis of a blood vessel. Three vessels were interrogated in each slice and then averaged. Averaged values were normalized to the signal maximum in the pial surface to allow comparison between animals.

## Results and Discussion

### Surgical considerations

The post-surgically induced inflammatory cascade resulted in transient opacity prohibiting TPFM within 24 to 48 hours, and up to 7 days, following cranial window implantation. Successful surgeries were evident two weeks after implantation of the cranial window, readily identifiable by unaided visualization of pial vessels and resolution of cortical bleeding or edema. Patency of the craniotomy was maintained following these successful surgeries for 12 weeks and longer. Two animals exhibited clear windows beyond 14 weeks (data not shown). Improvement in the window clarity was easily visualized throughout the time course of the experiment (Fig. 1A–D).

Size of the cranial window also affected the rate of wound healing. Smaller craniotomies (≤3 mm diameter) underwent rapid tissue healing by second intention. Large craniotomies (≥5 mm diameter) remained patent but sometimes led to distortion of the cortical parenchyma. A diameter greater than 5 mm required longer time for fibrotic scaffolds and fibroblasts to organize at the edge of the craniotomy. Since the removal of dura invariably promotes tissue swelling, small craniotomies maintain tissue architecture better. Large craniotomies provide multiple imaging sites within the window and facilitate assessment of spatial patterns in functional processes of interest. However, as size of the window increases, tissue disruption also increases. Six millimeter diameter window was found to be well suited for our experimental requirements. This size provided long term patency of the window, while still fitting over one cortical hemisphere.

Although the thinned skull method has allowed *in vivo* TPFM to cortical depths from 250 µm^2^ to 400 µm^1^ in the mouse, the thickness of rat meninges precludes microscopy of blood flow dynamics or neuronal activity below the pial surface. Tissue swelling may be controlled by gentle pressure on the glass coverslip, which helps re-establish intracranial pressure post cranial window implantation^7,33^. However, the thicker skull bone in the rat allows greater parenchymal swelling beneath the glass coverslip. To compensate, the skull around the craniotomy may be thinned to accommodate the coverslip placement closer to the pial surface^10^. Scott *et al*.^19^ devised a sophisticated chronic implant with the coverslip custom fit inside a metal ring, implanted to rest on the pial surface. This technique for reducing cortical swelling is customary in larger animals such as the rabbit^34^. Experimental criteria must be considered with each approach. Securing a coverslip adjacent to the pial surface may reinstate intracranial pressure yet provide a substrate for fibroblast migration along the inside surface of the glass coverslip into the centre of the craniotomy, or aggravate the pial surface. Conversely, we observed cementing the coverslip on the skull surface instigated healing from the window’s circumference^35^.

Frequently, protocols call for the application of agarose on the cortical surface to eliminate air bubbles trapped under the glass coverslip and to re-instate intracranial pressure^3^. Kwik-Sil (World Precision Instruments, USA), a silicon adhesive for securing implants, has been also suggested for TPFM imaging^5^. In our study, agarose appeared to irritate clotted blood vessels exposed in the bone or meninges during surgery. As the agarose cured, micro-bleeds were observed within the gelatin matrix^13^. These prevented endogenous cerebral spinal fluid from clearing the window. Agarose also appeared to increase opacity over time, distort the shape of the cortical surface in some of our rats, and provide a substrate for infiltration of fibroblasts. However, certain experimental protocols may require the use of such an interface when securing electrophysiological probes or other devices. For example, we used 1% agarose in an earlier study so as to stabilize the glass coverslip during the placement of a custom designed stainless steel miniature microscope base over the chronic window^36^. A novel application using silicone (polydimethylsiloxane) instead of a glass coverslip to close the window, provided competent TPFM over many weeks in the mouse, and also accommodated micro-injections or electrophysiology electrodes in the rat^20^.

Of particular importance for the clarity of the implant is minimization of time spent in surgery and thus under anesthesia^37^ as well as duration of cortical exposure to room air^16^. In particular, exposure of the brain to room air has immediate effects on cortical physiology, inducing an increase in arterial CO_2_ tension, alterations in vascular tone (vasodilation), promotion of microglial recruitment, leukocyte extravasation, and upregulation of reactive oxygen species^16,38–40^.

It is also critical to protect the cranial implant from inadvertent damage in the home cage. Standard housing of rodents on climate controlled ventilated racks is incompatible with the present procedures. Modified, custom-built, or commercially available enriched housing designs should be implemented. All overhead obstructions must be eliminated^7^ including wire tops dispensing food and water bottles, and hard plastic tubes or huts provided as environmental enrichment (Fig. 1F). Enrichment alternatives (e.g. autoclaved Nyla bones for gnawing, paper nesting material) must comply with animal management strategies defined by each facility. To further support the well-being of our rats longitudinally, cage mates were maintained in the same co-housed arrangement throughout the experiment. To avoid potential post-surgical aggression between dominant-submissive cage-mates, all co-housed rats underwent the cranial implant or sham surgery on the same day, recovered in a separate warming cage, and were then reunited in their original home cage. This ensured each cage-mate retained their relative social status thereby minimizing antagonistic behaviour.

### Neuro-inflammatory response

Inflammation is an inherent by-product of any surgical procedure resulting in activation of innate pro-inflammatory^39^ and systemic immune responses^41^. The type, magnitude, and duration of these is proportional to the magnitude of the tissue insult^18,30,41–43^. Heat, vibration, and pressure from drilling through the skull damages fragile bone matrix and underlying meningeal tissues. In addition, anesthesia itself can mobilize granulocytic leukocytes and T-cells^41^. This compromises the astrocytic end-foot barrier in the glial limitans^40^, elicts adjacent microglial pro-inflammatory cytokine release^39^, and may cause capillary thrombosis or micro-bleeds throughout the cranial window^15^. The primary inflammatory phase ensues over the first 48 hours with the pro-inflammatory activation of microglia, recruitment of systemic macrophages, and edema. The secondary phase initiates anti-inflammatory coordination for angio- and neuro-genesis, scarification, and tissue remodelling^39^ potentially leading to chronic tissue damage.

**Figure 2:**
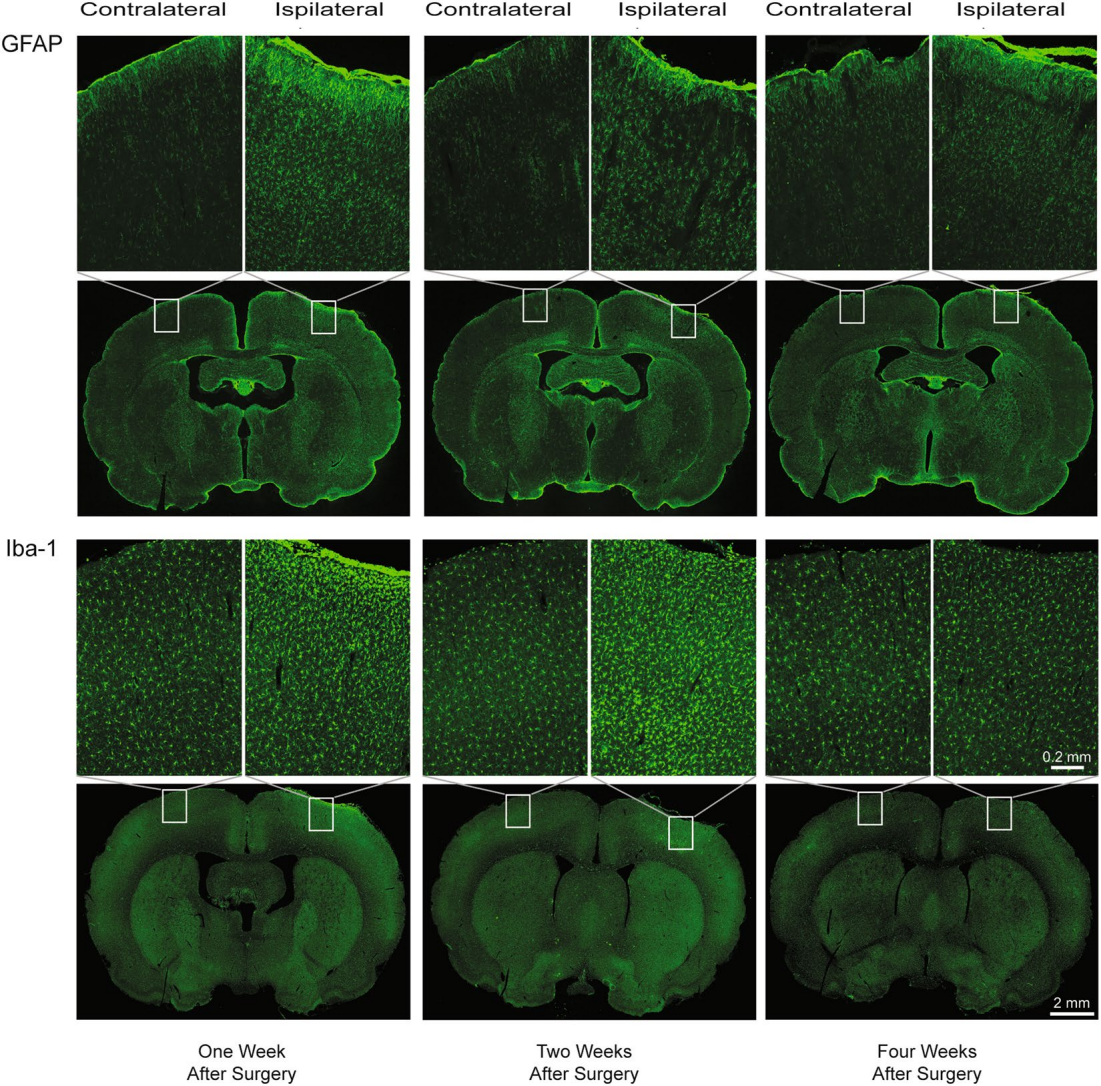
Immunofluorescence of glial fibrillary acidic protein (GFAP; top two rows) and ionized calcium-binding adaptor molecule-1 (Iba-1; bottom two rows). Coronal sections were obtained one week (left column), two weeks (middle column), and four weeks (right column) after surgical implant of the chronic craniotomy. Alternating coronal sections from a rat at each time-point were divided between GFAP and Iba-1 for processing. Coronal sections were selected around the mid-point of the craniotomy. The magnified inset for each coronal section illustrates the ipsilateral (right hemisphere; craniotomy), compared with the contralateral (left hemisphere; control) cortical region. The inflammatory pattern across time parallels the response demonstrated in mouse (Lagraoui *et al*.^42^) and in rat (Potter *et al*.^43^), following established wound healing sequences (Stroncek *et al*.^44^). GFAP shows astrocytic recruitment concentrated directly inferior to the craniotomy at one week, with more broadly spread cortical enhancement at two weeks, followed by attenuation by four weeks after surgery. Microglial/macrophage activation on Iba-1 demonstrate a similar spatial pattern of response along the pial surface adjacent to the craniotomy at one week, that continues to spread through the immediate cortical region at two weeks, then appears to resolve at four weeks after surgery. Scale bar: 2.0 mm coronal sections; 0.2 mm magnified inset.

Immuno-histological assessment of tissue response at one, two, and four weeks after the craniotomy demonstrates an early response near the pial surface, with gradual amelioration (Fig. 2). This inflammatory pattern is consistent with comprehensive reports assessing various intra-cranial procedures in the mouse^7,13,17,42^ and rat^18,43^, supporting frequently stated methods employing 2 to 4 weeks recovery after surgery. The time line corresponds with established wound healing progression^44^. At one week post-surgical implant, GFAP immunohistochemistry demonstrated astrocytic activation, recruitment and orientation towards the pial surface. The alteration in cellular arrangement was restricted within the cranial window region. Similarly, Iba-1 immunohistochemistry showed dense staining adjacent to the glial limitans, within the craniotomy. The microglial shape was ameboid rather than the normal ramified form observed during the resting state^39^. Two weeks post-surgical implant, GFAP and Iba-1 staining revealed spreading of the astrocytic and microglial/macrophage response into deeper cortical layers directly below the craniotomy. Microglial and astrocytic activation began to resolve by 4 weeks after surgery. Microglial populations proximal to the cranial window exhibit similar morphology and abundance between the ipsilateral (craniotomy) and contralateral hemispheres. On the other hand, astrocytes on the ipsilateral side display higher GFAP intensity, retracted processes, and denser distribution, characteristic of glial scarring (Supplementary Fig. S1). We did not observe any immuno-staining distal to the chronic craniotomy with either GFAP or Iba-1, suggesting strictly localized responses.

### Two-photon fluorescence microscopy

**Figure 3:**
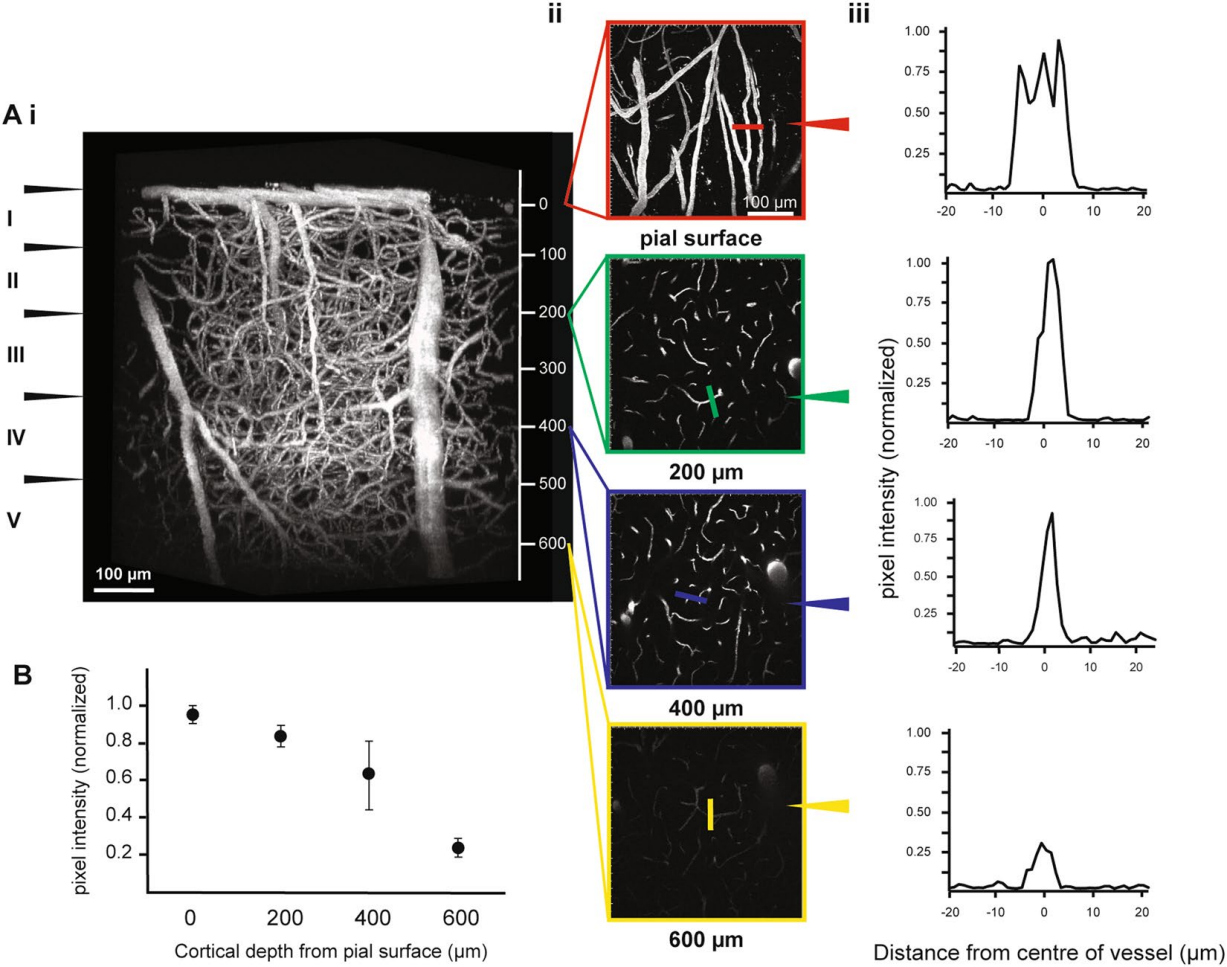
Image quality at 10 weeks after the craniotomy implant. Three-dimensional reconstruction from a two-photon fluorescence image of the cortical vasculature. (**Ai**) Maximum intensity projection (MIP) of cortical vascular architecture in a single rat to a depth of 660 µm below the pial surface. Estimated arrangement of the anatomical layers in the cortex is indicated in roman numerals along the left side (Yusufogullari *et al*.^45^). Scale bar: 100 µm. (**Aii**) Corresponding axial slices at the pial surface, 200 µm, 400 µm, and 600 µm cortical depths. Scale bar: 100 µm. (**Aiii**) Signal intensity profile across a single blood vessel randomly selected in each axial slice. Pixel intensity was normalized to the brightest pixel in the pial surface slice. Colour line on the axial slice indicates the location of the line on the chosen vessel. (**B**) The mean and standard deviation of pixel intensity normalized to the brightest pixel on the pial slice for 0 µm, 200 µm, 400 µm, and 600 µm cortical depths. Three vessels parallel to the imaging plane were chosen in each axial slice from all rats acquired at 10 weeks after the craniotomy. The vessels sampled within each slice were averaged and then normalized for comparison between rats.

A three-dimensional reconstruction of the vascular architecture at 10 weeks post surgical implant allowed visualization of cortical penetrating arteries and veins, as well as arterioles, capillaries and venules. Figure 3Ai illustrates the depth and fluorescent signal achievable in a single rat. Excellent intra- vs. extra-vascular contrast was observed in Layer I, encompassing 80–100 µm below the pial surface, Layer II/III 200–580 µm, and finally reaching the superficial region of Layer IV around 600 µm below cortical surface^45^ (Fig. 3Aii). Using progressive increases in laser intensity with depth, the signal-to-background ratio (SBR) remained relatively constant until 400 µm in cortical depth (Fig. 3Aiii). Of note, during most imaging sessions, we found the primary factor limiting imaging depth to be the working distance of the objective, in that the objective lens would come in contact with the glass coverslip or surrounding cement before the image contrast would disappear. Sampling three vessels in all rats imaged at 10 weeks revealed increasing variation between animals in the pixel intensity as depth reached 400 µm (Fig. 3B).

**Figure 4:**
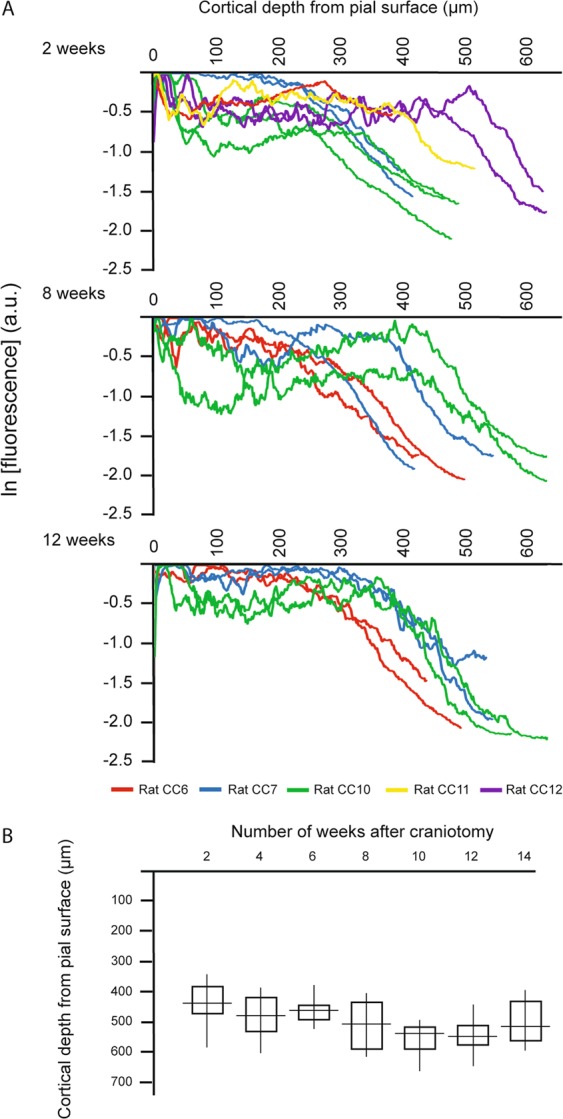
Variation between rats in the imaging signal profile and depth across time. (**A**) Fluorescent signal intensity versus cortical depth at 2, 8, and 12 weeks after the craniotomy implant comparing each image stack acquired The signal represents the mean signal intensity across each slice, normalized to the brightest pixel’s signal intensity. The imaging field-of-view was jittered at each time-point, although no damage was observable visually at the end of any imaging session. One to three fields-of-view were acquired from each rat, at each time-point: all data from a rat are shown in the same colour. The fields-of-view were randomly chosen at different sites within the cranial window, and were not necessarily repeated at subsequent time-points. Fluorescence a.u. – arbitrary units. (**B**) Comparison of the maximum cortical depth from the pial surface achieved at each time-point after the craniotomy, across all rats.

**Figure 5:**
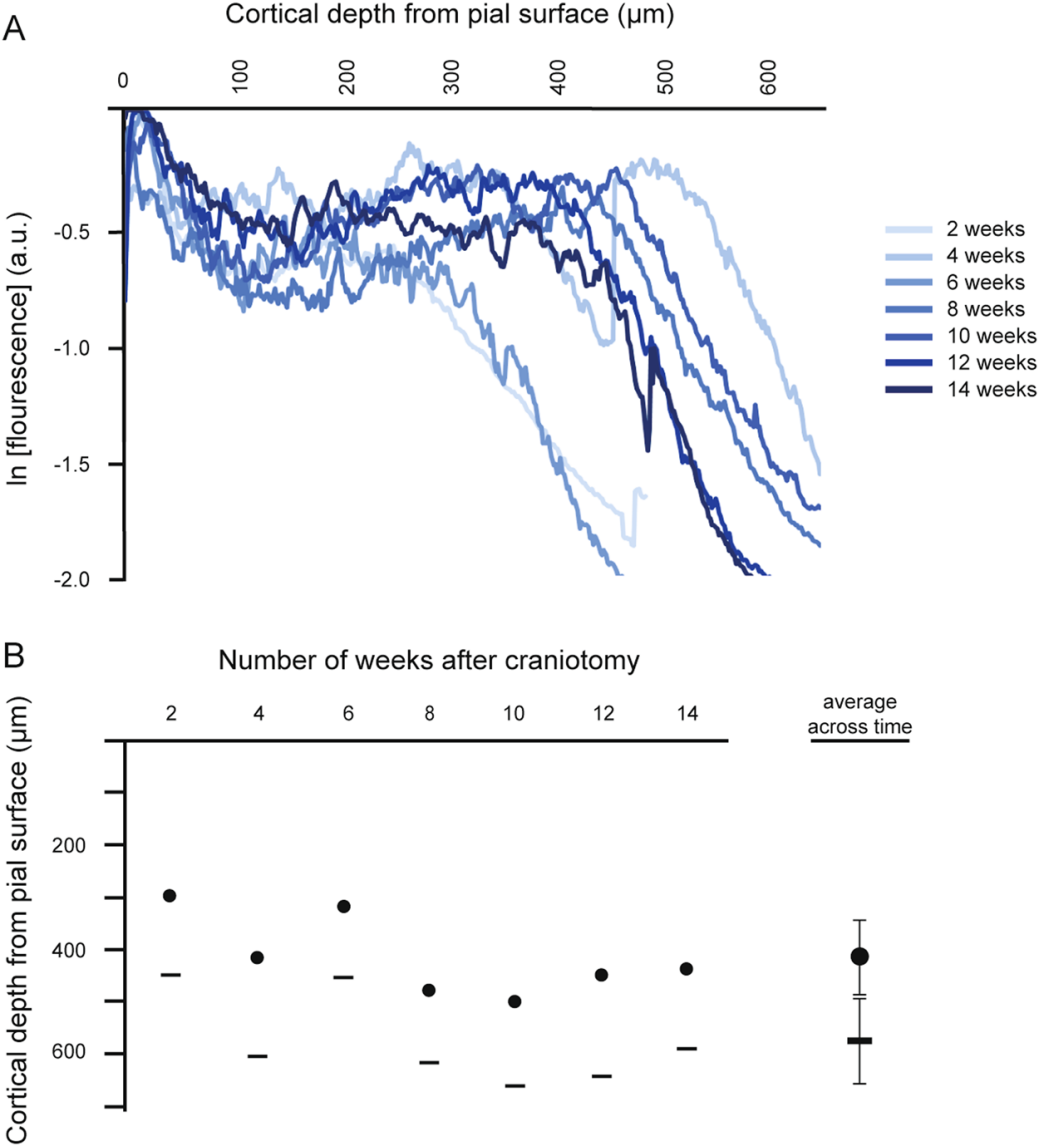
Variation of the imaging signal profile and depth within a single rat at each time-point after the craniotomy. (**A**) Fluorescent signal intensity versus cortical depth at each two week interval, between 2 and 14 weeks. The signal represents the mean signal intensity across each imaged slice, normalized to the brightest pixel’s signal intensity. Within the cranial window, two sites were selected at random for imaging, at each time-point. The data collected from these two fields-of-view were averaged to provide an estimate of signal decay with depth at that time-point. Fluorescence a.u. – arbitrary units. (**B**) Comparison of the cortical depth at signal intensity level of e^−1^ of the maximum surface signal intensity and the maximum imaging depth achieved in the rat at each time-point. (• e^−1^; ━ maximum).

The stack series acquired at 2, 8, and 12 weeks post-surgery demonstrated signal drop with depth: these data are plotted as the signal intensity normalized to the brightest pixel’s signal level (i.e. the pial vessel signal intensity). There was a characteristic dip in fluorescence just below the pial surface (likely due to light absorption by hemoglobin in the RBCs of large pial vessels), with a rise in signal through layer II/III, followed by roughly a linear decline in layer IV towards 650 µm depth^46^ (Fig. 4A). This pattern was consistent for most sites randomly selected within the cranial window for each rat, and across time. The progression from 8 weeks to 12 weeks reflects our qualitative observations of progressively decreased variability between rats and improved imaging depth. In this study, imaging depths were most consistent across animals at 10 and 12 weeks (Fig. 4B). Comparison of signal intensity and decay rate within a single rat across time (Fig. 5A) illustrates the decrease in variation from earlier (2–6 weeks) to latter time points (8–14 weeks) (Fig. 5B).

The depth at which signal of the brightest 10% of the pixels decreased by e^−1^ fold, since e^−2^ fold depth^47^ wasn’t imageable in most cases due to the limited working distance of the lens, for each site captured within the cranial window, in all rats and at each time-point, is listed in Supplementary Table S3. The average depth for signal decline to e^−1^ ranged from 354.4 ± 61.4 µm to 457.0 ± 16.8 µm across the 14 weeks. Greatest depth at e^−1^ and least variation between rats was achieved at 10 and 12 weeks, 457.0 ± 16.8 µm and 428.0 ± 18.2 µm respectively. The minimum and maximum total imaging depths achieved across time followed the same pattern, reaching peak tissue penetration at 10 weeks, with a range of 500 µm to 661 µm below the pial surface. TPFM data acquired over the 14 week time course in three rats, illustrates consistency within a single rat such that the mean depth at e^−1^ for rat CC6 was 372 ± 22 µm, rat CC7 was 386 ± 19 µm, and rat CC10 was 436 ± 20 µm. The average difference in maximum imaging depth achieved between two selected sites within a single craniotomy was 44.0 µm, ranging between no difference (i.e. same maximum imaging depth was achieved in both sites), and 183 µm difference in depths.

### Other considerations

Most reports on surgical techniques emphasize the need for experienced and highly skilled surgeons^3,18^. Initial trials in acute preparations for TPFM will guide the novice surgeon to achieve consistent technique and effectively manage anesthesia, animal manipulations, use of surgical instruments and equipment, and imaging. Progression evolves towards sterile surgical technique with refinements specific to each experimental design (Supplementary Table S1). Familiarization with physiological parameters and their changes during anesthesia as well as surgical effect’s, enhances one’s ability to monitor the animals’ well-being. Basic knowledge of wound healing and the endogenous inflammatory cascade is especially important for data interpretation in chronic imaging protocols. The quality of surgical preparations in rodents is enhanced when micro-structure of tissue can be visualized with the aid of a surgical stereoscope. Moreover, maintaining a surgical suite including surgical stereoscope, anesthetic system with multiple gas mixtures, physiological monitoring devices such as rodent pulse oxymeter, dedicated autoclave, and up-to-date surgical instruments and drapes, greatly facilitates the aseptic surgical technique.

In addition to technical skill, thorough application of controls addressing effects of surgical intervention is frequently overlooked, yet is critical for data interpretation. For example, the selection of drill type, whether trephine or round engraver drill bit, may have differential influence on the magnitude of inflammatory response and the time course of specific cytokines. A trephine induced a greater cytokine inflammatory response immediately after surgery than did the drill bit, while the drill bit produced greater behavioural impairments until reinstatement of baseline parameters over a 2 week recovery period^17^. Consideration of such confounds in techniques is important for unmasking physiological mechanisms of interest.

Pharmacological treatment during surgery and post-surgical recovery includes antibiotics, anti-inflammatories, and analgesics. Chronic cranial window protocols emphasize the use of antibiotics and anti-inflammatories^3,7,17,19^. Antibiotics are beneficial for reduction in the secondary systemic inflammatory response that increases leukocytic presence in the brain parenchyma^41^. Dexamethasone or non-steroidal anti-inflammatory drugs (NSAID) such as carprofen (e.g. Rimadyl, Zoetis Canada Inc) also enhance recovery and ameliorate systemic inflammation^41^. An opiod such as buprenorphine (e.g. Temgesic, Indivior UK Limited) is a common analgesic recommended by veterinary research staff. Although effective for short-term pain alleviation, opioids can significantly reduce food and water intake and prolong behavioural recovery for days^48,49^. Opioids also lower the seizure threshold via mu-receptor agonistic mechanisms potentiating absence or myoclonic seizures in a dose dependent manner^50–52^. Opiods may also contribute to increased intra-cranial pressures^53^. NSAIDs should be used with discretion in older or hypertensive models since this class of drug may exacerbate ischemic or hemorrhagic events, and may enhance seizure susceptibility^54,55^. It was noted in some reports that NSAIDs were administered simultaneously with dexamethasone and/or buprenorphine. This is contraindicated since NSAIDs exert both anti-inflammatory and analgesic effects, increasing the risk of an overdose. Informed application of pharmacologic support for the well-being of the animal will circumvent undesired consequences. At the same time, careful consideration of pharmacokinetics and potential side effects should precede the use of any drug routinely employed, especially with due consideration for confounding effects on the experiment.

## Supplementary information

Supplementary Information
