# Supplementary material for: Refinement of a chronic cranial window implant in the rat for longitudinal in vivo two–photon fluorescence microscopy of neurovascular function
Source: Sci Rep. 2019 Apr 2;9:5499. doi: 10.1038/s41598-019-41966-9 (PMC6445076; doi:10.1038/s41598-019-41966-9)
Supplement: Supplementary file 1 — Supplementary Information [file 41598_2019_41966_MOESM1_ESM.docx]

**Refinement of a chronic cranial window implant in the rat for longitudinal *in vivo* two–photon fluorescence microscopy of neurovascular function**

**Margaret M. Koletar**^2*^**, Adrienne Dorr**^2^**, Mary E. Brown**^2^, **JoAnne McLaurin**^2,3^**, and Bojana Stefanovic**^1,2^

^1^Department of Medical Biophysics, University of Toronto, 610 University Avenue, Toronto, Ontario, Canada M5G 2M9

^2^Sunnybrook Research Institute, 2075 Bayview Avenue, Toronto, Ontario, Canada M4N 3M5

^3^Department of Laboratory Medicine and Pathobiology, University of Toronto, 1 King’s College Circle, Toronto, Ontario, Canada, M5S 1A1

***Corresponding Author:**

Margaret Koletar

2075 Bayview Avenue S6 36

Toronto, ON M4N 3M5

416-480-6100 x3394

mkoletar@sri.utoronto.ca

**SUPPLEMENTARY INFORMATION**

**Supplementary Figure S1:**


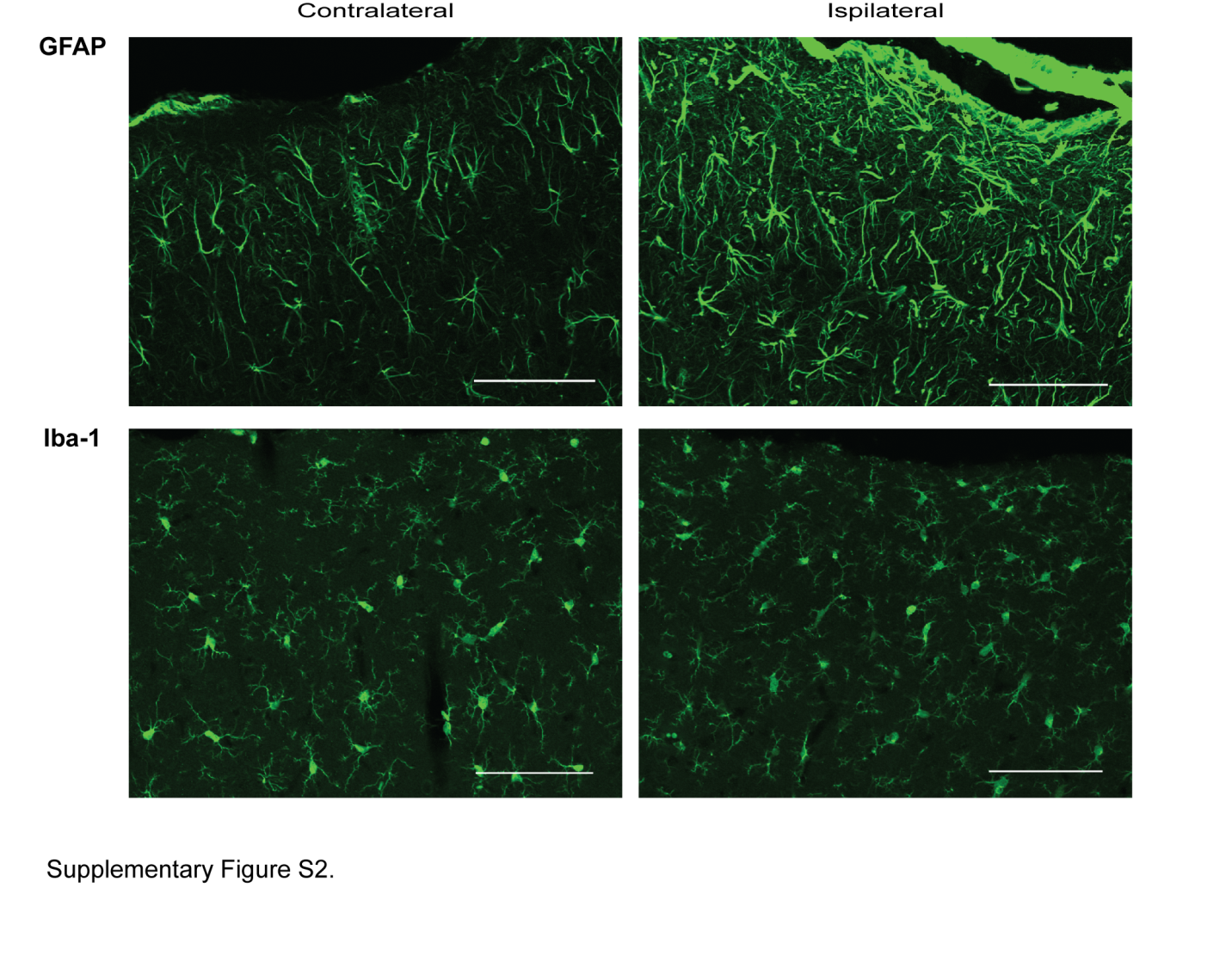


Higher magnification images of GFAP-positive astrocytes and Iba-1 positive microglia were taken from the same fields as that shown in Figure 2. Four weeks after the construction of the cranial window, microglial populations proximal to the cranial window exhibit similar morphology and abundance between the ipsilateral and contralateral hemispheres. On the other hand, astrocytes on the ipsilateral side display higher GFAP intensity, retracted processes, and denser distribution characteristic of glial scarring and astrocytic reactivity. Scale bar = 100 microns.

**Supplementary Table S1: Sequence for development of a successful chronic craniotomy in the rat**

| **Section** | **Timeline of the Development of a Chronic Craniotomy in the Rat** | **Goals** |
| --- | --- | --- |
| 1. Acute Chronic Craniotomy Surgeries for Two-Photon Fluorescence Microscopy  Timeline:  3 months or more, depending on previous experience and frequency of surgical practice. | The current protocol was developed by the Research Technician with professional experience in cell biology, physiology, and Veterinary clinical surgery and critical care; encompassing 6 years of stereotax experience prior to embarking on this project.  One year conducting stereotaxic surgeries in mice and/or rats for acute TPFM experiments, progressing to development of a chronic craniotomy in the rat. | - Prepare craniotomy in anesthetized rat with imaging immediately following completion of surgery. - Establish required or appropriate size and placement of cranial window to fulfill the experiment. - Cultivate atraumatic technique minimizing damage to tissues and reduce bleeding. - Improve and perfect dura removal minimizing cortical damage and inflammation. - Determine ideal medium for covering exposed pial surface; e.g. aCSF, saline, agarose. - Resolve ideal position of the glass coverslip, adhesive compound, and cement for accommodating the microscope objective lens. |
| 2. Development of Sterile Surgical Technique  Timeline:  1 month to 3 months (5 rats, or more as necessary) | Design and development of a chronic rat model specific for Laser Speckle Contrast Imaging (Sigal et al., 2016). 15 rats were used to design the cranial implant hardware and develop the rat model. Five more rats were required to refine the imaging technology. | - Assemble additional supplies required for sterile technique. - Prepare all supplies and instruments for sterilization. - Familiarization with additional surgical drugs and post surgical care. - Improve execution of sterile surgical technique and methods. - Refine skills utilizing surgical instruments, accessory equipment such as stereoscope, animal monitoring during and after anesthesia, etc. |
| 3. Pilot Study for TPFM with short term recovery time line.  Timeline:  1 to 3 months (5 to 10 rats) | Rat ID CC1 to CC5. Moderate success with clarity but limited depth for imaging. Refined details such as optimal craniotomy size, placement on the skull, adequate cementing, use of agarose versus saline/aCSF, improved skill to reduce tissue damage and inflammation. Animal housing and care was addressed to protect the cranial window. | - Progress to recovering rat after surgery, for 2 weeks. Monitor daily with assessment of cranial window clarity, patency, infection, fibroblast infiltration, damage from cage, etc. - Identify and resolve minor modifications or improvements to technique for specific experimental needs. - Perform post mortem assessment of craniotomy to ascertain and remedy issues from unsuccessful surgeries. - Conduct preliminary imaging at 1 and/or 2 weeks post surgery to gauge success of specific criteria in surgical technique. - Communicate with animal care staff for specific requirements; e.g. modifying cage arrangement to protect the cranial implant. |
| 4. Proceed to Experiment | Rat ID CC6 to CC12 continued with minor refinements to the technique and surgical skill.  CC6: Successful to 16 weeks. Cranial window TPFM imaging could have continued beyond this end-point.  CC7: Successful to 14 weeks with damage to cranial window at 16 weeks.  CC8: Died during 2 weeks post-surgery imaging.  CC9: Poor curing of cement well made TPFM imaging problematic. Chronic window was damaged at 6 weeks post-surgery.  CC10: Successful to 14 weeks. Cranial window TPFM imaging could have continued beyond this end-point.  CC11: Successful to 4 weeks. Complications with objective lens contacting cement well and damaging cranial window. Rat eliminated from experiments at 6 weeks post-surgery.  CC12: Successful to 4 weeks. Rat was transferred to another pilot study. | - Continue to develop and refine surgical skills. - Successful TPFM imaging of the chronic craniotomy at 2 weeks post-surgery increases likelihood of longitudinal imaging quality and data acquisition. |

**Supplementary Table S2. List of surgical supplies, and pharmacological agents described in this protocol.**

| **Surgical inventory of supplies** | | |
| --- | --- | --- |
| **Item** | **Source** | **Application** |
| Sterile surgical gown | Animal care facility: surgical department. | Provide covering to protect animal from infection during or after surgery. |
| Sterile drapes |  |  |
| Clean surgical cap, mask |  |  |
| Clean or sterile recovery cage |  |  |
| **Require sterilization prior to surgery:** | | |
| Fine rat tooth forceps | 11066-07 Fine Science Tools, Canada | Hold and retract skin. |
| Tissue forceps | 11006-12 Fine Science Tools, Canada | Hold and retract skin. |
| Iris scissors | 14106-09 Fine Science Tools, Canada | Cutting skin or other dense tissue. |
| Tissue retractor (Colibri) | 17000-3 Fine Science Tools, Canada | Separate skin for visualization of surgical site. |
| #7 Dumont forceps | 11297-00 Fine Science Tools, Canada | Fine manipulations of tissue. |
| #5 Dumont forceps | 11251-30 Fine Science Tools, Canada | Fine manipulations of tissue. |
| #5-angled Dumont forceps | 11251-33 Fine Science Tools, Canada | Fine manipulations of tissue. |
| Extra-fine spring scissors | 15003-08 Fine Science Tools, Canada | Durectomy (fine control to excise dura) |
| Mosquito haemostat | 13008-12 Fine Science Tools, Canada | Stop skin bleeding; to bend needle for dura incision. |
| Needle driver | 12002-12 Fine Science Tools, Canada | Hold suture needle to close skin. |
| Elevator / Spatula | 10089-11 Fine Science Tools, Canada | Apply dental cement. |
| Drill bits (0.45mm, 1.0mm) | Stoelting Co., USA | Drill skull bone. |
| 2”x2”, 4”x4” sterile gauze | Covidien, Medtronic , USA | Absorb fluids. |
| Kimwipes | Kimberly-Clark Worldwide Inc., USA | Absorb fluids. |
| Cotton swabs | Medline Industries Inc., USA | Clean skull surface |
| Glass beaker, 50 mL | Fisher Scientific, Canada | Cold saline/aCSF and gel sponge |
| Glass coversslips, 8mm | World Precision Instruments, USA | Cover and seal craniotomy. |
| **Other sterile supplies:** | | |
| Sterile surgical gloves | Encore, Ansell Healthcare, Canada | Provide covering of hands during surgery. |
| Scalpel, #10 blade | Bard-Parker, Aspen Surgical Products, USA | Initial skin incision. |
| Gelatin Sponge, Surgifoam | Ethicon Inc., USA | Extra-gentle absorption of fluids to be used with direct contact with dura/cortex. |
| 1mL, 3mL, and/or 5mL syringes | Becton-Dickenson Co., USA | Various injections, application of saline/aCSF during surgery. |
| 25 gauge needles | Becton-Dickenson Co., USA | Various injections, incision of dura. |
| 30mm sterile petri dish | Falcon, Corning Inc., USA | Preparation of dental cement. |
| 4-0 polysorb suture (SL5641) | Covidien, Medtronic, USA | Skin suture. |
| Saline (or artificial CSF) | JD7633 Baxter Corporation, Canada | Hydrate surgical site and flush debris to clear surgical site. |
| **General supplies:** | | |
| Cyanoacrylate glue (Lepage) | Henkel Corp, Canada | Securing glass coverslip to skull bone. |
| Tissue glue – Vetbond | 3M Animal Care Products, USA | Securing skin around cement in finished cranial window. |
| Anesthetic – Isoflurane | Fresenius Kabi Canada Ltd., Canada |  |
| Eye lubricant (Lacri-Lube) | Allercan Inc., Canada | Prevent dry eyes and corneal dessication. |
| Chlorhexidine soap | Atlas Laboratories Inc., Canada | Antibacterial soap to clean skin. |
| Isopropyl alcohol | Covidien, Medronic, USA | Antibacterial wipe to clean skin. |
| Iodine – Betadine Solution | Purdue Pharma, Canada | Antibacterial preparation to maintain clean skin and prevent infection. |
| 2”x2”, 4”x4” non-sterile gauze | Covidien, Medtronic, USA | Absorb fluids. |
| Kimwipes (non-sterile) | Kimberly-Clark Worldwide Inc., USA | Absorb fluids. |
| **Item** | **Source** | **Application** |
| **General equipment:** | | |
| Stereotax apparatus | Narishige Co. Ltd., Japan |  |
| Surgical table and lights | VWR International, Canada |  |
| Anesthetic vapourizor and system | Benson Medical Industries, Canada |  |
| Bead sterilizer | Germinator 500, Cell Point Scientific Inc., USA | To sterilize surgical instruments with the option for quick sterilization during surgery. |
| Weigh scale | Braintree Scientific Inc., USA | Body weight to calculate doses |
| Monitoring – MouseOx Plus | STARR Life Sciences Corp., USA | Pulse oxymetry monitoring during surgery. |
| Monitoring – Heating Pad | TC-1000, CWE Inc., USA | Maintain core body temperature during surgery. |
| Micromotor drill | Stoelting Co., USA |  |
| Surgical stereoscope | Tritech Research Inc., USA | Accurate and clear visualization during surgery. |
| Rodent electric shaver | Wahl Canada Inc., Canada |  |
| Heating pad (or lamp) | Sunbeam Products Inc., USA | Maintain core body temperature during post surgery recovery |

| **Pharmacological agents used intra- and post-surgery** | | | |
| --- | --- | --- | --- |
| **Type** | **Name** | **Dose** | **Application** |
| Anesthetic | Isoflurane | 5% induction; 1.5-2.5% maintenance | Facilitate real-time adjustments in the depth of anesthesia. |
| Analgesic | Buprenorphine | 0.05 mg/kg (subcutaneous) | Reduce post-surgical pain, stress-induced secondary inflammatory response, and minimize self-inflicted trauma to surgical site (e.g. scratching, grooming). |
| Antibiotic | Enrofloxacin | 5 mg/kg (subcutaneous) | Reduce acute infection at surgical site and moderate systemic inflammatory response (Denes *et al*.). |
| Anti-inflammatory | Dexamethasone | 0.1 mg/kg (intraperitoneal) | Reduce edema/swelling and moderate post-surgical secondary inflammatory response (Denes *et al*.). |
| Local Anesthetic | Marcaine | 7 mg/kg, administered subcutaneously on dorsal skull | Reduce local pain (skin, periosteum) and self-inflicted trauma to the surgical site (e.g. scratching, grooming). |
| Hydration agents | Lactate Ringers Solution or 0.9% physiologic saline | 3 mL (subcutaneous) | Alleviate dehydration due to anesthetic effects and assist elimination of metabolized intra-operative drugs. |
| Antibiotic ointment | Bioderm | As needed applied topically on the surgical site. | Topical treatment on the surgical incision site. Aids in wound healing. |
| Post-surgical analgesic | Carprofen*,* | 5 mg/kg (subcutaneous) | NSAID: Analgesic and anti-inflammatory. |

**Supplementary Table S3: Depth (µm) of the signal intensity at *e*^-1^ for each site acquired within a cranial window, at each time-point.**

|  | **Time-point: Number of weeks after surgical implant of the chronic craniotomy** | | | | | | | | |
| --- | --- | --- | --- | --- | --- | --- | --- | --- | --- |
| **Rat ID-FOV#** | **2 weeks** | **4 weeks** | **6 weeks** | **8 weeks** | **10 weeks** | **12 weeks** | **14 weeks** | **Mean Depth at *e*^-1^** | **SEM** |
| CC6-1 | na | 247.5 | - | 345.0 | 427.5 | 390.0 | 456.0 | 372.3 | ±21.5 |
| CC6-2 | - | 385.5 | - | 271.5 | 417.0 | 360.0 | 423.0 |  |  |
| CC7-1 | 313.5 | na | na | 420.0 | 462.0 | 471.0 | 295.5 | 385.9 | ±19.0 |
| CC7-2 | 334.5 | 388.5 | 450.0 | 318.0 | 426.0 | 435.0 | 316.5 |  |  |
| CC10-1 | 340.5 | 375.0 | 454.5 | 490.5 | 520.5 | 469.5 | 448.5 | 435.9 | ±20.1 |
| CC10-2 | 309.0 | 559.5 | 315.0 | 453.0 | 489.0 | 442.5 | 435.0 |  |  |
| CC11-1 | 411.0 | - | 198.0* | - | - | - | - |  | |
| CC11-2 | - | - | na* | - | - | - | - |  |  |
| CC12-1 | 534.0 | 454.5 | - | - | - | - | - |  |  |
| CC12-2 | 484.5 | - | - | - | - | - | - |  |  |
| **Mean Depth**  **at *e*^-1^** | 389.6 | 401.8 | 354.4 | 383.0 | 457.0 | 428.0 | 395.8 |  |  |
| **SEM** | ±33.8 | ±41.9 | ±61.4 | ±34.6 | ±16.8 | ±18.2 | ±28.9 |  |  |
| **Minimum Total Imaging Depth** | 351.0 | 391.5 | 376.5 | 406.5 | 499.5 | 445.5 | 390.0 |  |  |
| **Maximum Total Imaging Depth** | 579.0 | 603.0 | 520.5 | 613.5 | 660.0 | 643.5 | 589.5 |  |  |

The TPFM imaging depth (µm) at which the decay in signal intensity reached *e*^-1^, varied both at the first time-point and across time. In general, imaging depth improved from 2 weeks to 8 weeks post-surgery, peaked by 10 weeks, then indicated a decline after 14 weeks post-surgery. Successful image acquisition at 2 weeks post-surgery generally indicated sustained imaging quality to 14 weeks or more. There was greater variation between rats in a single time-point, than for a single rat across all time points (Rat CC6, CC7, and CC10). Differences between two sites randomly selected within the cranial window at a single time point varied (mean 44.0 µm; range 0.0 µm to 183.0 µm) depending on the tissue characteristics at the chosen site, space within the window to accommodate the objective lens, or perpendicular alignment achieved between the objective lens to cortical surface.

ID-FOV: rat identification number and field-of-view

SEM: standard error of the mean

na: The signal intensity remained above *e*^-1^ for the total depth of TPFM image acquisition.

“-“: Data was not acquired.

“*”: Technical complications with TPFM laser function compromised image acquisition.
